# Study of correlation between wall shear stress and elasticity in atherosclerotic carotid arteries

**DOI:** 10.1186/s12938-017-0431-y

**Published:** 2018-01-16

**Authors:** Bo Zhang, Junyi Gu, Ming Qian, Lili Niu, Dhanjoo Ghista

**Affiliations:** 1 Department of Ultrasound in Medicine, Shanghai East Hospital, Tongji University School of Medicine, Shanghai, 200120 China; 2 Paul C. Lauterbur Research Center for Biomedical Imaging, Shenzhen Institutes of Advanced Technology, Chinese Academy of Sciences, Shenzhen, 518055 China; 3 University 2020 Foundation, Northborough, MA 01532 USA

**Keywords:** Texture matching method, Atherosclerosis, Arterial elasticity, Wall shear stress, Carotid artery

## Abstract

**Objective:**

This paper presents the use of the texture matching method to measure the rabbit carotid artery elasticity value of the experimental group and control group respectively. It compares the experimental rabbits, when they are prompted by pathological histology to be at the period of carotid atherosclerosis fatty streaks and fiber plaques, with the control group.

**Methods:**

We have used ultrasound linear array probe for scanning the rabbit carotid arteries. This allows us to obtain the wall shear stress (WSS) and the elasticity values in the atherosclerotic arteries. Using statistical analysis, we are able to clarify whether the texture matching method can diagnose atherosclerosis at the early stage. We also analyze the rabbit carotid artery elasticity and WSS values to make sure whether there is a correlation between both. Combining the texture matching method with the WSS quantitative analysis in the future can enable better prediction of the occurrence and development of atherosclerosis by using noninvasive medical imaging techniques.

**Results:**

This study has confirmed that from the 2nd to the 10th week, with the development of atherosclerosis, the arterial WSS reduction has a negative correlation with the increasing of artery wall elasticity, which means that as the arterial WSS decreases the arterial wall becomes less elastic. Correlating shear stress with atherosclerosis can clarify that WSS can be used as one of the effective parameters of early diagnosis of atherosclerosis.

**Conclusion:**

In summary, we have found that the elasticity value can reflect the degree of atherosclerosis more objectively. Therefore, by using noninvasive imaging, the quantitative analysis of shear stress and combined with texture matching method can assist in the early diagnosis of atherosclerosis.

## Background

Cardiovascular diseases have a high mortality rate in China [1], and the key cause of cerebral and myocardial infarction is atherosclerosis [2, 3]. The formation of atherosclerosis is a long and complicated process. In general, atherosclerosis is divided into four types, which are fatty streaks, fibrous plaque, atheromatous plaque, and complicated lesions [4]. Sometimes, there has been evidence of atherosclerosis, but there are no specific clinical symptoms of atherosclerotic stenosis [5], which means that stenosis is not the critical factor in evaluating atherosclerosis. The intima-media thickness can be used to check on atherosclerosis [6, 7]. However, other schools of thoughts have insisted that this has a limited indication for early diagnosis and prediction of atherosclerotic related diseases [8, 9]. Now, wall shear stress (WSS) is known to affect atherosclerosis [10–17], such that the morphology and function of the vascular endothelium is influenced, which can stimulate the migration and proliferation of endothelial smooth muscle cells and mononuclear cells [18]. Low and oscillating WSS is often used as an index of causation of atherosclerotic plaque [19], which can deem it to be a reliable indicator for evaluating atherosclerosis [20, 21]. A quantitative analysis software for WSS measurements can be implemented for such quantitative analysis [22], whereby the Hagen–Poiseuille formula can be used to compute WSS changes [23]. Some scientists believe that the WSS is inversely correlated to blood lipid [24]. In particular, low WSS values causes atherosclerotic lesions, and this may be due to flow separations [25].

Arterial elasticity refers to the features of the arterial blood vessel whereby its blood vessel volume will change as the pressure inside the vessel changes, which is also called arterial compliance. Arterial elasticity can hence be used for the diagnosis of atherosclerosis. Arterial elasticity changes can often be detected in early stages of atherosclerosis [26].

Medical imaging modalities exist to perform cardiac diagnosis [27–30]. Intravascular ultrasound and common ultrasound are the common methods of measuring arterial elasticity. Intravascular ultrasound has high image resolution, but pertains to an invasive examination. It is expensive, and presents surgical risk. The ultrasound is easy to operate, but the image resolution is limited. Clinical ultrasound is typically implemented for measuring intima-media thickness, which allows medical experts to be able to determine if they should intervene clinically [31]. We note that although WSS is an important and useful factor in determining the occurrence and development of atherosclerosis [32], other parameters could well be used, such as elasticity measurements of arteries. Now, there are many functional parameters required to evaluate arterial elasticity clinically, such as pulse wave velocity, bulking index, compliance, stiffness, bulking index and elastic modulus value, etc. In this regard, clinical trials show that the elastic modulus value can better reflect the changes of arterial elasticity. Hence, in this study, the elastic modulus value is selected to be the evaluation index of arterial elasticity [33]. Higher elastic modulus value is associated with lower arterial elasticity.

In this study we use the texture matching method, which is a blood vessel elasticity imaging technology based on two-dimensional ultrasound images. This technology, which is based on using the texture matching analysis of a series of continuous ultrasonic two-dimensional images collected by ultrasonic imaging system, obtains a blood vessel wall’s 2-dimensional displacement vector map at different times in a cardiac cycle, and finally obtains distribution of 2-dimensional elastic modulus of blood vessel walls through calculating the displacement vector map. Based on the blood vessel’s 2-dimensional elastic modulus distribution, we can distinguish the composition distribution of the walls, the collagen fibers, elastic fibers and smooth muscle cells [34].

## Methods

### Experimental animals and grouping

In our experimental study, we employed a total of 60 healthy white male New Zealand rabbits (provided by Shanghai Tongji University Animal Laboratory), which are approximately 10 weeks old and weighed 2–2.5 kg. By use of a randomized method, they were divided into two groups: 20 in normal control group and 20 in the experimental group. Ethics on animal experiments was approved by the institutional board of Tongji University.

### Instruments used

The Philips IE33 Diasonograph (Philips Medical Systems, Andover, MA, USA) as well as high frequency probe L15-7 (Philips Medical Systems, Andover, MA, USA) were used in our study.

### Pharmaceutical application

We applied Atropine Sulfate injection, 0.5 mg/ml (developed by Shanghai Wellhope Pharmaceutical Co. Ltd., and approved by H31021172). Then, for Ketamine hydrochloride injection, we implemented 2 ml at 0.1 g (by Jiangsu Hengrui Pharmaceutical Co. Ltd., and approved by H32022820).

### Animal models

Experimental rabbits were fed with high-fat feedstuff, which was bought from Trophy Feedstuff Technology Co. Ltd. (where feedstuff code is TP2R118), and caused to develop atherosclerosis artificially. These New Zealand rabbits in the experimental group were fed with the feedstuff at 50 g/kg/day once every 12 h, and allowed to drink water with no restraint for a total of 10 weeks. The temperature of breeding environment was controlled at around 15 °C, and the rooms were kept ventilated and clean in accordance with animal ethnics. We implemented the experimental rabbit intramuscular anesthesia with ketamine hydrochloride (22 mg/kg) and atropine sulfate (70 μg/kg) mixture [35].

### Measurement of rabbit blood pressure

**Fig. 1:**
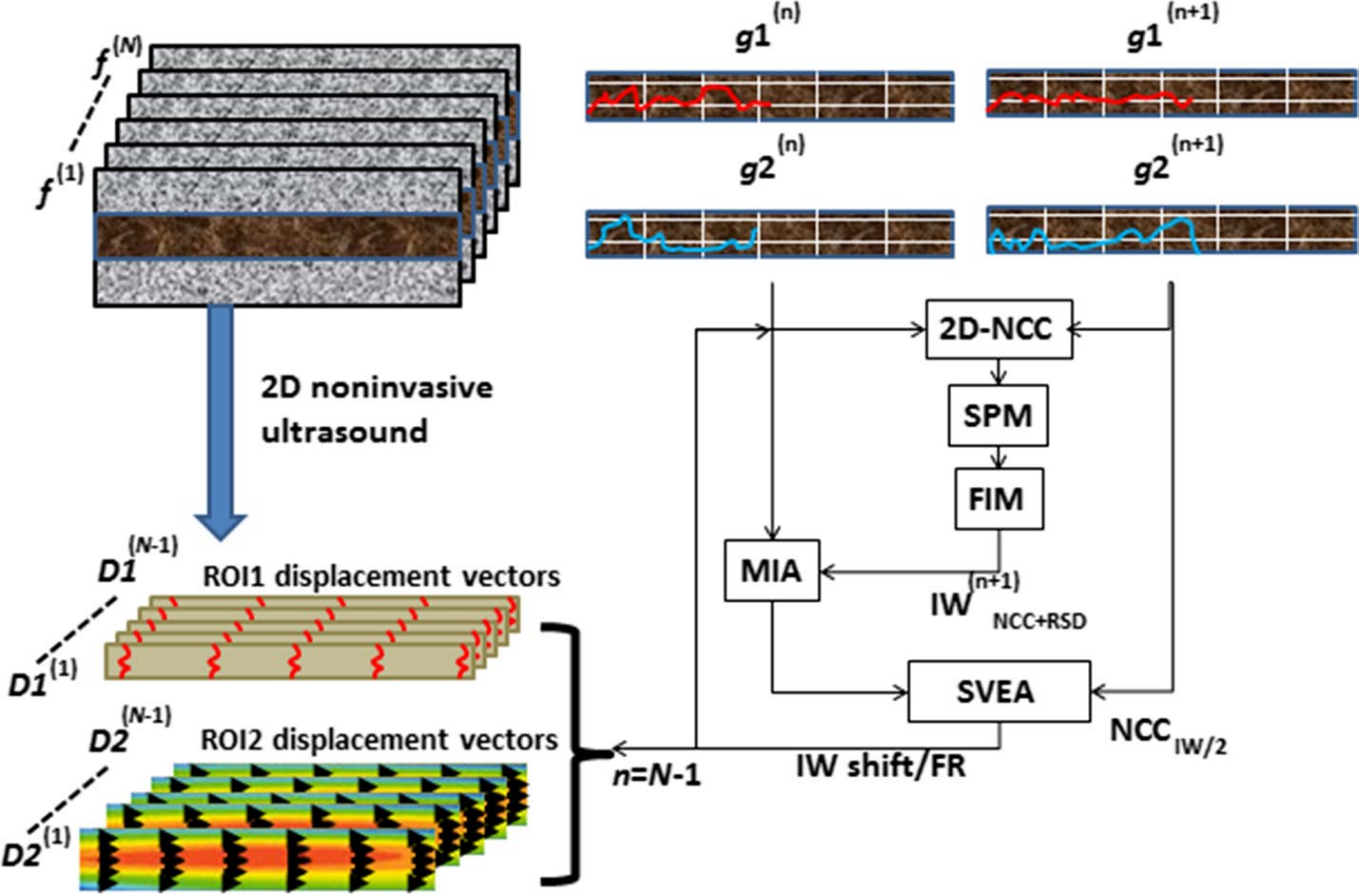
The principle of texture matching method based on a flowchart illustrating how the arterial elasticity value can be computed

Next, we performed the following procedures: (1) connect the artery to the blood pressure detecting device (pressure energy transducer); (2) fill the casing system with heparin saline; (3) weigh the rabbits; (4) make the experimental rabbit intramuscular anesthesia with ketamine hydrochloride (22 mg/kg) and atropine sulfate (70 μg/kg) mixture. (5) fix the rabbits on the operating table, supine, after anesthesia and shear the neck hair. (6) Cut open the neck skin and extract the trachea and carotid artery; (7) conduct tracheotomy and insert Y-shape tube, and use a thick thread to ligate; (8) use thick thread to ligate distal arteries and Clamp the proximal part of the common carotid artery with vascular clamp (with a distance of 1.5 cm); (9) snip the proximal part and insert a casing pipe (connected to RM6240 biological signaling system). (10) Take off the artery clamp after tightening the proximal part. (11) Finally, start signal collection and record the changes in rabbit blood pressure. Note that the key procedural steps are illustrated in Fig. 1.

### Wall shear stress quantitative analysis

We used the Philips IE33 diasonograph, L15-7 linear array probe for scanning the rabbit carotid arteries in the longitudinal section, and we ensured that the ultrasonic cross-section passes through the center axis of the blood vessels. The acoustic beam was directed at 60° angle to the common carotid artery. We adjusted (i) the speed range to make the lumen full of blood flow without aliasing, and also (ii) the sampling frame range, in order to keep the Doppler graphics frame frequency between 20–30 frames. Then, we collected the Doppler blood flow images of the common carotid artery, about 1–2 cm below the mandibular angle plane, in both the experimental group and the control group. The images were saved in the DICOM format. These images are able to allow us to determine the WSS using a numerical formula by transforming the color images representing Doppler blood flow velocity into velocity data, according to the maximum speed and the color indication range.

### Texture matching method

**Fig. 2:**
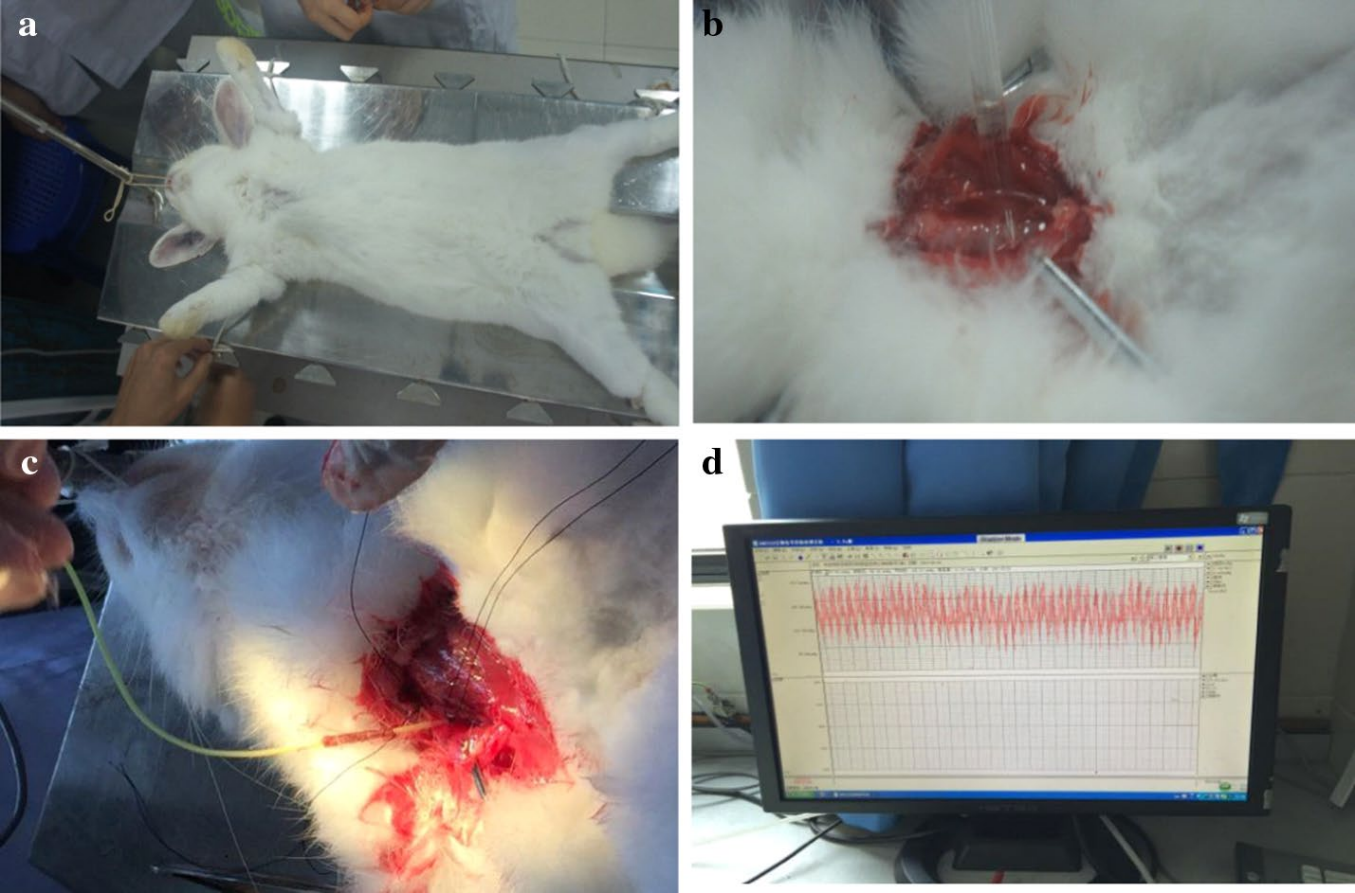
The following procedures are performed based on **a** fixation of the rabbit model on the operation table; **b** extraction of rabbit common carotid artery; and **c** connection of casing pipe to the rabbit common carotid artery; and **d** rabbit blood pressure measurement

Through statistical analysis, we are able to clarify whether the texture matching method can diagnose atherosclerosis at the early stage. The texture matching method is presented in Fig. 2.

#### Image acquisition of texture matching method

Using the same instrument, Philips IE33 diasonograph, L15-7 linear array probe, we connect the electrocardiogram (part of diasonograph) to the rabbit’s two forelegs and left hind leg, and observe the electrocardiographic changes of the experimental rabbit. Under the condition of stable rabbit heart rate, we scan the rabbit common carotid artery in the longitudinal section, and adjust the gain to make the rabbit carotid artery wall intima-media clearly shown. We also adjust the sampling frame range and keep image frame rate between 190–230 frames. We continuous record the dynamic images of CCA longitudinal section, about 1–2 cm below the mandibular angle plane, in three cardiac cycles of the experimental group and control group. The images are saved in DICOM format, as mentioned previously.

#### The analysis process of texture matching method

The following steps are carried out: (1) the image is divided into multiple analysis windows; (2) use of 2-dimensional standard cross-correlation algorithm, sub pixel method and the filtering interpolation method on the analysis window to get the 2-dimensional displacement (translation) of the vessel wall; (3) repetitive iterative algorithm uses the displacement gradient of the translation to compensate for the rotation and deformation of the blood vessel walls; (4) the 2-dimensional standard cross-correlation algorithm uses small analysis window for high spatial resolution, and uses error vector algorithm to get displacement estimation of high-precision; (5) conduct the above operation on all analysis windows in the entire frame of images to get the 2-dimensional displacement vector field of the frame; (6) repeat the above process, and get several displacement vectors of each layer of vascular wall caused by the heart throb at different times in the cardiac cycle.

### Statistical processing

SAS9.3 software was used in statistical analysis. Measurement data is described with range given by ± s. Comparison among groups uses the *t* test. We use the Pearson correlation analysis to determine the correlation between the rabbit carotid artery elasticity and WSS of the experimental group. Note that P < 0.05 means that the difference has a statistical significance.

## Experimental results

### Histopathologic examination

Based on observation, the structures of arterial intima, tunica media and tunica externa are complete. The internal elastic membrane is continuous. No intimal thickening and no foam cells are seen beneath the intima.

### Analysis of the common carotid artery elasticity

There are statistical differences in *t* test between the two groups from the 8th week onwards. It suggests that the rabbit carotid artery elasticity level of the experimental rabbits is higher than that of the control group in the 8th week and the 10th week (Table 1).

**Table 1:** The rabbit carotid artery elasticity value pertaining to both the experimental group and control group (kPa)

Group type	Week 2	Week 4	Week 6	Week 8	Week 10
Experimental group	44.95 ± 14.6	47.76 ± 12.8	60.62 ± 20.4	87.52 ± 46.8	162.14 ± 97.8
Control group	39.10 ± 17.2	41.62 ± 15.2	50.52 ± 16.8	55.80 ± 19.1	54.95 ± 22.4
P value	0.253	0.175	0.096	0.001	0.001

Conduct *t* test on the rabbit carotid artery elasticity values of the experimental group and the control group. Here, P values < 0.05 in the 8th week and 10th week. We observe statistical differences in the two groups

### Columnar analysis diagram of rabbit carotid artery elasticity value

**Fig. 3:**
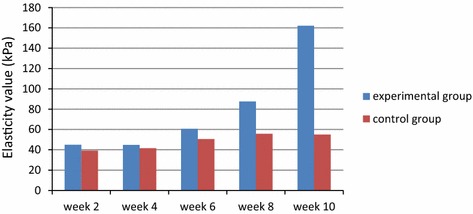
Dynamic variation diagram of rabbit carotid artery elasticity value between the experimental group and the control group

The rabbit carotid artery elasticity value of the experimental group has increased with clear observation after the 8th week (Fig. 3).

### Common carotid artery elasticity value

**Fig. 4:**
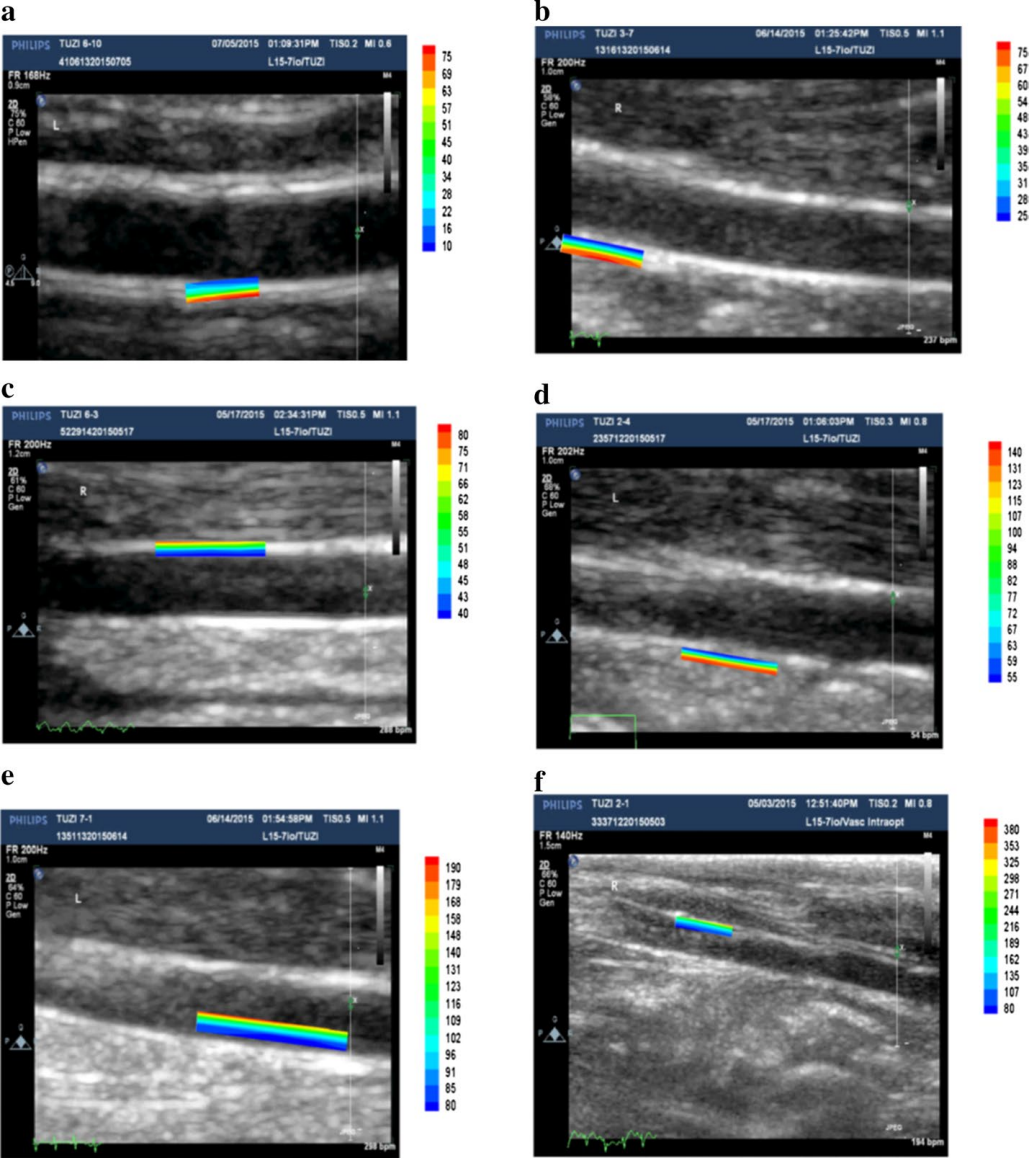
The texture matching method used in analysis of rabbit carotid artery elasticity value based on **a** control group; **b** experimental group (2 weeks); **c** experimental group (4 weeks); **d** experimental group (6 weeks); **e** experimental group (8 weeks); and **f** experimental group (10 weeks)

The common carotid artery elasticity value increases with the increasing degree of arteriosclerosis (Fig. 4). In the sub-figures as shown: (a) the rabbit carotid artery elasticity value of control group, with interval of 10–75; (b) rabbit carotid artery elasticity value of experimental group (2 weeks), with interval of 25–75; (c) rabbit carotid artery elasticity value of experimental group (4 weeks), with interval of 40–80; (d) rabbit carotid artery elasticity value of experimental group (6 weeks), with interval of 55–140; (e) rabbit carotid artery elasticity value of experimental group (8 weeks), with interval of 80–190; and (f) rabbit carotid artery elasticity value of experimental group (10 weeks), with interval of 80–380.

### Correlation between Elasticity value and WSS value

**Fig. 5:**
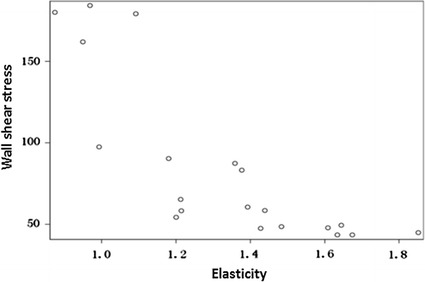
A negative correlation for the wall shear stress versus the elasticity can be observed in the form of a scatter diagram

The relevance between common rabbit carotid artery elasticity value of the experimental group and WSS value is discussed here. Based on Pearson correlation analysis the elasticity value is negatively correlated to WSS. Here, *r* = 0.8, *P* < 0.01 (see Table 2). The Pearson correlated coefficient, wall shear stress, and elasticity values are examined by using a scatter plot in Fig. 5.

**Table 2:** The correlation between rabbit carotid artery elasticity value and the WSS value whereby the Pearson correlation analysis results in correlated coefficient r = 0.8, P < 0.01, and show negative correlation

Pearson correlation analysis Prob > |r| under H_0_: Rho = 0		
	Wall shear stress	Elasticity
Wall shear stress	1.00000	− 0.80302 (P < 0.0001)
Elasticity	− 0.80302 (P < 0.0001)	1.00000

## Discussion

Arterial elasticity can reflect the characteristics of the vascular wall elasticity. As an index for diagnosis of cardiovascular disease, it gets more and more attention in clinical use. Arterial elasticity is mainly determined by the tunica media in the arterial wall. When pathological changes of atherosclerosis occur, we see tunica media smooth muscle cells and collagen fibre hyperplasia in great quantities. The vessel wall’s elastic membrane disrupts, the arterial elasticity drops and the arterial elastic modulus value rise accordingly.

The vascular elasticity imaging technology provided by texture matching method can conduct vascular wall elasticity images in real-time and non-invasively, and obtain the distribution of the 2-dimensional elastic modulus of blood vessels. It can classify components of the blood vessel wall, and distinguish the distribution of collagen fibers, elastic fibers and smooth muscle cells in the of blood vessel walls [36]. Some scholars have used the texture matching method to measure the rat carotid artery wall elasticity and have obtained an average elasticity (or elastic modulus) value of 134.62 ± 54.3 kPa. After the formation of atherosclerosis plaque inside the blood vessel wall, the plaque of different ingredients can be divided into: lipid (81 ± 40 kPa), blood clots (95 ± 56 kPa), fibrous tissue (1.0 ± 0.63 MPa), and calcification tissue (2.0 ± 1.2 MPa). As confirmed by using the method of texture matching, there are significant differences between the rat carotid artery elasticity value of the normal group and the group with atherosclerosis [37].

In this study, before the 8th week, there was no significant difference between the rabbit carotid artery elasticity of the experimental group and the control group (P > 0.05). Even in the 6th week, when the control group is confirmed by pathological histology to be in the period of rabbit carotid artery fatty streaks, because the pathological histology found elastic membrane integrity of rabbit carotid artery tunica media and no obvious thickening in the tunica media (Table 1), there is no statistical difference between the rabbit carotid artery elasticity value of the experimental group and control group.

However at the 8th weeks and 10th week of this study, statistical differences are found in rabbit carotid artery elasticity of the experimental group compared with the control group (P < 0.01) (Table 1). The pathological histology found a large quantity of smooth muscle cells and collagen fibre hyperplasia in the tunica media of rabbit carotid arterial wall, and the arterial elasticity (or elastic modulus) value rose correspondingly (see Figs. 3, 4).

Because the lipid elasticity value is similar to the normal artery elasticity value, there is no hyperplasia in the smooth muscle cells and collagen fiber in the arterial wall tunica media, or no destruction of elastic membrane; and therefore, for the lipid period of atherosclerosis, the elasticity value is not a sensitive indicator. When atherosclerosis arises during the formation of fiber plaques and because of a large number of hyperplasia of smooth muscle cells and collagen fiber, there is higher content of fibrous tissue and the arteries gradually harden, with corresponding changes in the arterial elasticity (modulus) value.

Traditional ultrasonic diagnosis method of atherosclerosis is through the morphology, structure and changes of echo blood vessels of the vascular wall, while the texture matching method can make quantitative diagnosis without relying on vascular wall morphology changes. It can reduce man-made interference, and reflect the degree of atherosclerosis more objectively. Therefore, when the elasticity value rises and there is statistical difference compared with the control group, it means that atherosclerosis comes into the period of fiber plaques.

This study has confirmed that from the 2nd to the 10th week, with the development of atherosclerosis, arterial WSS reduction has a negative correlation with the increasing of artery wall elasticity (Fig. 5), which means that as the arterial WSS reduces the artery becomes less elastic. Correlating shear stress with atherosclerosis can clarify that WSS can be used as one of the effective parameters of early diagnosis of atherosclerosis. In this study on vessel wall elasticity and atherosclerosis, we have found that the elasticity (modulus) value can more objectively reflect the degree of atherosclerosis. So under the condition of noninvasive methodology, the quantitative analysis of shear stress combined with texture matching method can work in the early diagnosis of atherosclerosis, and can get us specific shear stress values for evaluation. Shear stress values have high sensitivity and specificity for the analysis of the degree of atherosclerosis, and can become one of the indexes of determining the degree of atherosclerosis.

## Conclusion

Under the condition of noninvasive methodology, the texture matching method can more objectively reflect the degree of hardening of the arteries, and combined with quantitative analysis of blood flow shear stress, can diagnose the occurrence of early atherosclerosis along with specific shear stress values. It can offer help to clinical experts in the early intervention and treatment of atherosclerosis. Two important findings are as follows:

1. The occurrence of cardiovascular and cerebrovascular disease is correlated to arterial plaque rupture. The part of the artery plaque that comes into the lumen will change the geometry of the arteries and is bound to cause blood flow shear stress changes. The nature of the plaque itself also has a close relationship with plaque rupture. The combination of quantitative analysis of blood flow shear stress with analysis of the arterial elasticity using the texture matching method can be used in the research of the relativity of arterial plaque rupture.
2. The study sample size is not sufficiently large, which may make the studies on rabbit carotid arterial WSS and arterial elasticity insufficient. We will enlarge the sample size in future research, to improve the early diagnostic accuracy of the carotid artery atherosclerosis by using WSS and wall elasticity.

## References

[1] Zhang P (2012). Long-term exposure to ambient air pollution and mortality due to cardiovascular disease and cerebrovascular disease in Shenyang, China. PLoS ONE.

[2] Mas JL (1998). Prevention of cerebral infarct caused by atherosclerosis. Arch Mal Coeur Vaiss.

[3] Tu JY (2011). Analysis of patient-specific carotid bifurcation models using computational fluid dynamics. J Med Imaging Health Inform.

[4] Sarkar RN (2014). Adult onset Still’s disease with persistent skin lesions complicated by secondary hemophagocytic lymphohistiocytosis. Int J Rheum Dis.

[5] Karim R (2008). Relation of Framingham risk score to subclinical atherosclerosis evaluated across three arterial sites. Am J Cardiol.

[6] Frerix M (2014). Atherosclerotic plaques occur in absence of intima-media thickening in both systemic sclerosis and systemic lupus erythematosus: a duplexsonography study of carotid and femoral arteries and follow-up for cardiovascular events. Arthritis Res Ther.

[7] Molinari F, Zeng G, Suri JS (2010). A state of the art review on intima-media thickness (IMT) measurement and wall segmentation techniques for carotid ultrasound. Comput Methods Programs Biomed.

[8] Diener HC, Sacco R, Yusuf S (2007). Cerebrovascular diseases. Cerebrovasc Dis.

[9] Bassetti C, Aldrich MS (1999). Sleep apnea in acute cerebrovascular diseases: final report on 128 patients. Sleep.

[10] Roger VL (2001). Time trends in the prevalence of atherosclerosis: a population-based autopsy study ☆. Am J Med.

[11] Targonski P (2001). Referral to autopsy: effect of antemortem cardiovascular disease: a population-based study in Olmsted County, Minnesota. Ann Epidemiol.

[12] Wong KKL (2012). Biomechanical investigation of pulsatile flow in a three-dimensional atherosclerotic carotid bifurcation model. J Mech Med Biol.

[13] Wong KKL (2012). Effect of calcification on the mechanical stability of plaque based on a three-dimensional carotid bifurcation model. BMC Cardiovasc Disord.

[14] Cheung SCP (2010). Experimental and numerical study on the hemodynamics of stenosed carotid bifurcation. Australas Phys Eng Sci Med.

[15] Wong KKL (2010). Modelling of blood flow resistance for an atherosclerotic artery with multiple stenoses and poststenotic dilatations. ANZIAM J E.

[16] Wong KKL (2006). Theoretical modeling of micro-scale biological phenomena in human coronary arteries. Med Biol Eng Comput.

[17] Wong KKL, Mazumdar JN, Abbott D. A study of the relationship between geometrical variation of atherosclerotic arteries and flow resistance. In: Proceedings of the International Federation for Medical and Biological Engineering and the 12th International Conference on Biomedical Engineering (12th ICBME 2005), Singapore, 2005. Vol.12. p. 3A5-01.

[18] Böyum A (1968). Isolation of mononuclear cells and granulocytes from human blood. Isolation of monuclear cells by one centrifugation, and of granulocytes by combining centrifugation and sedimentation at 1 g. Scand J Clin Lab Invest Suppl..

[19] Ross R (1999). Atherosclerosis—an inflammatory disease. N Engl J Med.

[20] Hays AG (2012). Regional coronary endothelial function is closely related to local early coronary atherosclerosis in patients with mild coronary artery disease: pilot study. Circ Cardiovasc Imaging.

[21] Senior RM (1982). Elastase of U-937 Monocytelike cells: comparisons with elastases derived from human monocytes and neutrophils and murine macrophagelike cells. J Clin Invest.

[22] Zhang L (2014). Quantitative blood flow shear stress analysis software in evaluation on carotid atherosclerosis. Chin J Medical Imaging Technol.

[23] Uramoto H, Yamada S, Tanaka F (2013). Angiogenesis of lung cancer utilizes existing blood vessels rather than developing new vessels using signals from carcinogenesis. Anticancer Res..

[24] Zhou (2017). Effects of metformin on blood pressure in nondiabetic patients: a meta-analysis of randomized controlled trials. J Hypertens.

[25] Davies PF (2009). Hemodynamic shear stress and the endothelium in cardiovascular pathophysiology. Nat Clin Pract Cardiovasc Med.

[26] Cachovan M (1969). Changes of some parameters of arterial elasticity in man during early atherosclerosis of the lower limbs. Angiology.

[27] Wong KKL (2009). Cardiac flow analysis applied to phase contrast magnetic resonance imaging of the heart. Ann Biomed Eng.

[28] Wong KKL (2010). Cardiac flow component analysis. Med Eng Phys.

[29] Wong KKL (2009). Medical imaging and processing methods for cardiac flow reconstruction. J Mech Med Biol.

[30] Wong KKL (2013). Methods in research and development of biomedical devices.

[31] Cheng RQ, et al. The Correlation Research on Ba PWV and IMT of Patients with Carotid Atherosclerosis. Henan Traditional Chinese Medicine. 2015.

[32] Tascilar N (2009). Relationship of apoE polymorphism with lipoprotein(a), apoA, apoB and lipid levels in atherosclerotic infarct. J Neurol Sci.

[33] Messas E, Pernot M, Couade M (2013). Arterial wall elasticity: state of the art and future prospects. Diagn Interv Imaging.

[34] Ushiki T (2002). Collagen fibers, reticular fibers and elastic fibers. A comprehensive understanding from a morphological viewpoint. Arch Histol Cytol.

[35] Vidal CJ. Ketamine hydrochloride. Revista Española De Anestesiología Y Reanimación. 1970;17(2). 5490504

[36] Niu LL (2012). A texture matching method considering geometric transformations in noninvasive ultrasonic measurement of arterial elasticity. Ultrasound Med Biol.

[37] Bézie Y (1998). Fibronectin expression and aortic wall elastic modulus in spontaneously hypertensive rats. Arterioscler Thromb Vasc Biol.

